# Supplementary material for: Iterative improvement in the automatic modular design of robot swarms
Source: PeerJ Comput Sci. 2020 Dec 7;6:e322. doi: 10.7717/peerj-cs.322 (PMC7924708; doi:10.7717/peerj-cs.322)
Supplement: Supplemental Information 3 [file peerj-cs-06-322-s003.zip › argos3/doc/api/standalone/a00563_source.html]

ARGoS: plugins/robots/spiri/simulator/spiri\_entity.h Source File


- Main Page
- Related Pages
- Namespaces
- Classes
- Files

- File List
- File Members

# plugins/robots/spiri/simulator/spiri\_entity.h

Go to the documentation of this file.

```
00001 
00007 #ifndef SPIRI_ENTITY_H
00008 #define SPIRI_ENTITY_H
00009 
00010 namespace argos {
00011    class CControllableEntity;
00012    class CEmbodiedEntity;
00013    class CSpiriEntity;
00014    class CQuadRotorEntity;
00015    class CRABEquippedEntity;
00016    class CPerspectiveCameraEquippedEntity;
00017 }
00018 
00019 #include <argos3/core/simulator/entity/composable_entity.h>
00020 
00021 namespace argos {
00022 
00023    class CSpiriEntity : public CComposableEntity {
00024 
00025    public:
00026 
00027       ENABLE_VTABLE();
00028 
00029    public:
00030 
00031       CSpiriEntity();
00032 
00033       CSpiriEntity(const std::string& str_id,
00034                    const std::string& str_controller_id,
00035                    const CVector3& c_position = CVector3(),
00036                    const CQuaternion& c_orientation = CQuaternion(),
00037                    Real f_rab_range = 3.0f,
00038                    size_t un_rab_data_size = 10,
00039                    const CRadians& c_cam_aperture = ToRadians(CDegrees(30.0f)),
00040                    Real f_cam_range = 10.0f);
00041 
00042       virtual void Init(TConfigurationNode& t_tree);
00043       virtual void Reset();
00044 
00045       inline CControllableEntity& GetControllableEntity() {
00046          return *m_pcControllableEntity;
00047       }
00048 
00049       inline CEmbodiedEntity& GetEmbodiedEntity() {
00050          return *m_pcEmbodiedEntity;
00051       }
00052 
00053       inline CQuadRotorEntity& GetQuadRotorEntity() {
00054          return *m_pcQuadRotorEntity;
00055       }
00056 
00057       inline CRABEquippedEntity& GetRABEquippedEntity() {
00058          return *m_pcRABEquippedEntity;
00059       }
00060 
00061       inline CPerspectiveCameraEquippedEntity& GetPerspectiveCameraEquippedEntity() {
00062          return *m_pcPerspectiveCameraEquippedEntity;
00063       }
00064 
00065       virtual std::string GetTypeDescription() const {
00066          return "spiri";
00067       }
00068 
00069    private:
00070 
00071       CControllableEntity*              m_pcControllableEntity;
00072       CEmbodiedEntity*                  m_pcEmbodiedEntity;
00073       CQuadRotorEntity*                 m_pcQuadRotorEntity;
00074       CRABEquippedEntity*               m_pcRABEquippedEntity;
00075       CPerspectiveCameraEquippedEntity* m_pcPerspectiveCameraEquippedEntity;
00076    };
00077 
00078 }
00079 
00080 #endif
```

---

Generated on 10 Jul 2018 for ARGoS by 
 1.6.1
